# Data-Driven Machine Learning Calibration Propagation in A Hybrid Sensor Network for Air Quality Monitoring

**DOI:** 10.3390/s23052815

**Published:** 2023-03-04

**Authors:** Ivan Vajs, Dejan Drajic, Zoran Cica

**Affiliations:** 1 School of Electrical Engineering, University of Belgrade, 11120 Belgrade, Serbia; 2 Innovation Center of the School of Electrical Engineering in Belgrade, 11120 Belgrade, Serbia; 3 DunavNET, DNET Labs, 21000 Novi Sad, Serbia

**Keywords:** air quality, air pollution monitoring, low-cost sensors, hybrid network, machine learning, sensor calibration, calibration propagation

## Abstract

Public air quality monitoring relies on expensive monitoring stations which are highly reliable and accurate but require significant maintenance and cannot be used to form a high spatial resolution measurement grid. Recent technological advances have enabled air quality monitoring that uses low-cost sensors. Being inexpensive and mobile, with wireless transfer support, such devices represent a very promising solution for hybrid sensor networks comprising public monitoring stations supported by many low-cost devices for complementary measurements. However, low-cost sensors can be influenced by weather and degradation, and considering that a spatially dense network would include them in large numbers, logistically adept solutions for low-cost device calibration are essential. In this paper, we investigate the possibility of a data-driven machine learning calibration propagation in a hybrid sensor network consisting of One public monitoring station and ten low-cost devices equipped with NO_2_, PM_10_, relative humidity, and temperature sensors. Our proposed solution relies on calibration propagation through a network of low-cost devices where a calibrated low-cost device is used to calibrate an uncalibrated device. This method has shown an improvement of up to 0.35/0.14 for the Pearson correlation coefficient and a reduction of 6.82 µg/m^3^/20.56 µg/m^3^ for the RMSE, for NO_2_ and PM_10_, respectively, showing promise for efficient and inexpensive hybrid sensor air quality monitoring deployments.

## 1. Introduction

It is estimated that about 55% of the global population is currently situated in cities, with an ever-increasing trend, and there are predictions that in the year 2050 the percentage will be as high as 68% [1]. In addition to social, infrastructural, traffic, and energy consumption issues that will pose significant challenges, the level of air pollution in cities will also be affected by the increase in various pollution emitters. As a result, there will be an increase in the number of urban zones with high levels of air pollution, which could have a strong influence on the citizens’ health and result in adverse health outcomes (chronic obstructive pulmonary disease, heart attacks, etc.) [1]. Heavy urban traffic congestion and air pollution represent key health challenges for cities worldwide.

The main goal of air quality monitoring in urban areas is to detect and understand pollution sources and trends in areas of interest. In [2], the specific goals of air quality monitoring are defined as follows: compliance reporting against the Ambient Air Quality Directives, information for the public, identifying long-term trends in concentrations, elaboration of air quality plans, and assessment of the effectiveness of abatement measures. Additionally, the environment-type classification of measuring sites is provided in [2] as follows: urban, suburban, rural, background, traffic, and industrial. In urban air quality monitoring, the pollution conditions could be observed at different scales, therefore requiring an adequate definition of monitoring use cases. In one of the urban monitoring categorizations [3,4], monitoring locations are categorized as micro-scale locations (to 0.1 km), middle-scale locations (0.1–0.5 km), neighborhood scale locations (0.5–4 km), and urban scale locations (4–50 km), while types of monitoring sites are categorized as curbside monitoring, roadside monitoring, and urban background monitoring.

When taking into consideration the need to monitor specific city areas, the urban environment suffers from an insufficient number of public monitoring stations. This is due to their size, price and the fact that their locations are fixed. The most promising solution to overcome this issue is the additional deployment of a number of low-cost devices (thus making a low-cost sensor network) which will improve the detection of the sources of pollution and personal exposure. They are easy to deploy (small size) and mount (for example: a solid object, pillar, or wall), have wireless communication modules for real-time data transmission, and can be installed in practically any needed location. The devices should be placed at a height ranging from 1.5 to 4 m (in the human breathing zone). These low-cost devices could be used to collect complementary measurements and to support public monitoring stations by increasing spatial and temporal measurement resolution [3,4].

The selection of locations should be made in such a way as to avoid measurements being taken in the immediate vicinity of the source of pollution. Devices should be placed at least ten meters from the edge of main traffic intersections, and one hundred meters from any possible pollution source. When deploying monitoring set-ups, several factors should be considered, including the topography of the monitored area, distance from the expected sources of pollution, available mounting locations, existing obstacles, etc.

Additionally, this provides an excellent benchmark for the creation of city pollution maps. Pollution maps are dynamic maps that should be updated on an hourly/bi-hourly basis since terrain structure, buildings’ environment, traffic data, and other meteorological data (such as wind) also influence their characteristics. In order to create accurate enough measurements for pollution maps and identify pollution hot spots, it is necessary to create a dense enough network of monitoring devices but still consider the cost-effectiveness in order to ensure accessibility to a large number of cities.

Devices with low-cost sensors have their drawbacks, particularly with measurement accuracy and sensor calibration. The most intuitive way to overcome the shortcomings of both public monitoring stations and low-cost devices, is their combined deployment, thus creating a “hybrid sensor network” where public monitoring stations are expanded by the usage of multiple low-cost devices. This kind of deployment allows for a much easier process of periodic sensor recalibration without the need to physically collect the low-cost devices and bring them into the laboratory or collocate them with a public monitoring station for the purposes of the recalibration process. In this hybrid sensor network, the recalibration will be achieved by correlating the low-cost device with the closest public reference monitoring station, or by cross-calibration with the nearest recalibrated devices in the hybrid sensor network area. Furthermore, public monitoring stations will gain the support of complementary measurements, which are obtained by using spatially distributed low-cost devices.

Obviously, massive use of low-cost sensors/devices along with a low number of expensive public monitoring stations is a promising approach toward the expansion of air quality measurement coverage and pollution map creation. Thus, in Section 1.1 the work related to air quality monitoring solutions that are based on low-cost devices is presented. In Section 1.2, the goals and contributions of the research study proposed in this paper are presented.

### 1.1. Related Work

In the literature, there are various proposals and scenarios regarding the deployment of a sensor network for air quality monitoring and for conducting a calibration and recalibration process. First, we will discuss solutions based on low-cost devices that do not consider calibration and recalibration aspects. Then, we will discuss solutions that consider calibration and recalibration aspects. Most of existing proposals are based on mobility: either a calibrated device is moving and used in calibration/recalibration of non-moving devices, or non-calibrated devices are moving and are calibrated when in vicinity of a calibrated station. The large number of recent papers that address these topics indicate the importance of an economic approach to air-quality measurement solutions. Finally, we will present our previous research that is relevant to the study results presented in this paper.

In [5], an analysis of the network with six low-cost PM sensors deployed in Southampton, in two schools and a referent station, (which is 1 km and 2 km distant from the devices) was performed. The collected results during a seven-month campaign were compared with the reference data, and promising results were obtained (in the sense of result trends and correlation with the reference results), but recalibration is not considered (although correlation degradation is obvious after some time). In [6], the authors deployed 24 low-cost air quality devices across Oslo, Norway to monitor NO_2_ pollution for three months with the goal of creating air quality maps. The data fusion method was proposed and evaluated, and it showed that data collected from low-cost devices together with reference monitoring stations could extract valuable information for the development of urban air quality maps. However, sensor recalibration was not taken into consideration. The authors in [7] deployed 40 sensor devices at London Heathrow Airport with the goal of monitoring and distinguishing airport emissions from long transport emissions. The sensors were first validated by comparing them with the reference monitoring station at the airport. The analytical approach was defined and used during a five-week campaign to calculate ratios of the airport activities in different locations of the airport. However, the sensors’ recalibration process was not considered. In [8], the authors obtained data from a sensor network consisting of 126 low-cost sensor devices deployed in Nanjing, China. Data obtained from 13 existing reference stations from the same area were used for the validation of the performance of low-cost devices. The devices were divided into clusters based on the respective reference stations, and validation methods were proposed for accuracy, reliability, and failure detection. By verifying performance, they concluded that low-cost sensor networks could be a valuable solution for air quality monitoring, but that in-field calibration and recalibration models should be applied to improve the accuracy of the measurement. In the paper [9], the authors proposed and described a new rapid deployment method for low-cost sensor deployment (in Taiwan) that consisted of the following phases: preparation, implementation, and modification. First, basic input data were defined (objectives, spatial data locations, elimination rules). Then in the next phase, information about the desired deployment density and algorithm settings were inserted. Finally, in the modification phase, the user could redefine the deployment density and again evaluate the results. The obtained result was the recommended number of sensor and deployment locations. Depending on the use case, they proposed a predefined sensor density; for example, for industry and traffic areas, a radius of 300 m per sensor is recommended (a sensor is placed at every 300 m), while for areas without heavy sources of pollution, the radius is assumed to be 500–1000 m (the user could change these default values). However, calibration and recalibration issues were not considered.

Most of the proposed recalibration schemes are based on mobile deployments, where calibrated (in this case, reference), low-cost devices are mounted on vehicles, thus providing virtual references when these sensors enter the vicinity of low-cost target devices. In [10], the authors deployed a network of ten low-cost devices in fixed locations in Bari, Italy, and one mobile device (installed on a public bus) was used to expand the measurement coverage. Data analysis covered a period of 30 months. Results were compared to public reference monitoring stations, and it was concluded that the usage of low-cost sensors provided promising results for achieving the data quality objective of the indicative measurements [2]. On the other hand, they concluded that for long-term sensor operation, recalibration is of crucial importance. In [11], the authors used devices installed on vehicles and wearable devices to calibrate static sensors (in Zurich, Switzerland) which will, later on, calibrate sensors in their vicinity, thus creating a multi-hop calibration where calibration is propagated through an array of sensors. However, this kind of calibration is only effective in preserving data consistency if the calibration error is not accumulated at each hop. In order to reduce this error accumulation, the authors proposed a sensor array network calibration using a multi-dimensional linear regression technique. In [12], the authors proposed a novel algorithm to reduce error propagation in multi-hop calibration systems (deployed in Zurich, Switzerland) which showed better performance than the ordinary least squares method. In [13], the authors elaborated on a massive-scale air quality monitoring network by deploying tens of thousands of sensors for air quality monitoring with fine spatial resolution on the testbed deployment case in Helsinki. The authors proposed the usage of vehicles with mounted low-cost devices with recently calibrated sensors to pass by other devices in order to propagate air quality and calibration information. Experimental results showed performance improvements, but it was concluded that in order to ensure that recalibration would capture reference patterns as much as possible from the limited dataset, machine learning techniques would be necessary. In [14], the authors observed a mobile air quality monitoring system (Mosaic project, devices are mounted on buses [15] in Hangzhou and Ningbo, China) and proposed a two-phase data calibration method that consisted of a linear and a nonlinear part. For the linear part MLS (multiple least square) training was used, while for the nonlinear part, random forest (RF) training was used. The method was verified on PM_2.5_ measurements and claimed an improvement of accuracy by 16.4% in comparison to linear models. In [16], a hybrid sensor network architecture was proposed that consisted of mobile and stationary devices for indoor air quality monitoring to measure inter-zone air flow, since pollutant concentrations can vary notably even within the same monitoring building. Mobile sensors were carried by people in order to measure personal exposure data. For sensor accuracy correction and improvement, the Bayesian analysis-based solution was proposed. They have developed a model for predicting the pollutant level and have defined algorithms for hybrid network deployment in a building. In general, approaches with mobile deployments of devices show promising results, however, due to the way data were collected, these solutions were generally lacking from the perspective of data quantity, since for reliable calibration it is necessary to correlate data over a longer period of time with a higher temporal resolution.

In [13], the authors proposed a so-called opportunistic and collaborative recalibration method, wherein a device with a low-cost sensor collected calibration information whenever an opportunity arose, i.e., when the device is located close enough to a reference station. This concept is taken further in [17], where the authors proposed an In-field Calibration Transfer (ICT), i.e., a calibration method that transfers the calibration parameters of reference sensors to target field stations in Beijing, China. The authors adopted a calibration transfer approach proposed in [18,19] where calibration transfer was defined as a calibration paradigm for sensors that did not have access to reference measurements (target sensors) by using sensors with reference values (source sensors). In [18,19], calibration transfer was conducted on electronic nose instruments, i.e., sensor arrays for hazardous odor detection, while in [20], sensor measurements of concentration levels of ethanol, ethylene, carbon monoxide, or methane were observed. In [18,19,20], calibrations of target sensors were performed by transferring calibration parameters from the source sensors. The method was proposed and proven with the idea to reduce the initial calibration overhead and calibration costs in mass production and development, i.e., to avoid collocation of sensors for initial calibration, while the authors [17] proposed an improvement of this method for the recalibration (in-field calibration) process. Namely, they used an RF algorithm to correct the measurements of devices that are collocated with reference stations and considered a calibration transformation function that was obtained by optimizing the Kullback-Leibler (KL) divergence between the probability distributions of calibrated and uncalibrated sensor measurements. This effectively removed the need for collocation or temporal synchronization, stating that when the probability distributions of measurements are similar between two sensors, the calibration can be transferred between them using a linear transformation function. They conducted experiments and concluded that ICT method was able to accurately calibrate the target sensors, by comparing the results of the calibration with available true reference stations.

Authors [21] analyzed the PM_2.5_ monitoring network deployed in Denver to evaluate different calibration models across the network. First, they evaluated 21 calibration models that included temperature (T), relative humidity (RH), and dew point (D) data; 16 models were multivariate regression calibration models (adapted from [22]) and 5 models relied on machine learning techniques. Root mean square error (RMSE) and Pearson coefficient r were used for evaluation. A detailed comparison was provided, and suggestions were given regarding which calibration model could be most useful for usage in a specific use case. As the next step, they evaluated calibration transfer possibilities from co-located referent device sites (five) to the rest of the network and statistically compared the errors in predictions for each device. Different corrections were applied based on an entire dataset correction, an on-the-fly correction, a two-week winter correction and a two week winter + two-week spring correction (for the first two proposed corrections, more complex calibration models showed better prediction performances). In conclusion, the authors claimed that some calibration models performed well but also could result in large errors at specific sites and that metrics developed in this paper could be used for the evaluation of transfer metrics.

In our previous work, we focused on the development of a methodology for the calibration of off-the-shelf air quality sensors [23]. The conducted calibration process was based on the use of statistical algorithms and offset values obtained from the reference measurement stations. The obtained results have shown that low-cost sensors could be used with a relatively high reliability as a complementary network to reference monitoring stations, however, it was also concluded that every sensor has its own sensitivity to temperature and relative humidity that influence the measurement accuracy. In [24], we explored methods to additionally improve the calibration algorithms with the aim to increase the measurement accuracy by considering the impact of temperature and humidity on the readings by using machine learning. A detailed comparative analysis of linear regression, artificial neural network, and random forest algorithms were presented, analyzing their performance on the calibration of measurements of CO, NO_2_, and PM_10_ particles, with promising results measured by achieved increase of correlation coefficient between the reference monitoring station and the low-cost device. Furthermore, the concept of a Hybrid Sensors Network Approach was proposed.

### 1.2. Objective and Contributions of the Study

In this paper, we will present a method for calibration transfer and evaluate it on a network of 10 low-cost sensors and a single reference station. We observed NO_2_ and PM_10_ measurements during two different months and evaluated a concept of calibration propagation through the sensor network. As previously described, there are many papers that consider hybrid sensor networks and that discuss the possibilities of sensor calibration transfer. Most of these solutions use mobility to achieve collocation between a non-calibrated device and a reference station. However, in many cases, vehicles used can be pollution sources (for example, buses) which affects the measurements, and thus, the calibration/recalibration process. The method proposed in this paper, however, focuses on a different manner of calibration transfer. The proposed calibration focuses on the density of a sensor network and machine learning algorithms. The devices are stationary (not mobile) and can be placed in proper locations that are not in the immediate vicinity of pollution sources. With this method, there is no need for laboratory usage, which could be expensive, or mobile monitoring stations and occasional collocation periods with a reference station, which could suffer from insufficient collocation data. Thus, our solution is very economic and does not suffer from occasional collocation, but the collocation is permanent. The study proposes a “multi-hop” calibration concept where calibration is transferred through a chain of low-cost devices from a single reference station. Since there is no mobility of low-cost devices and collocation is permanent, our solution is able to use acquired data for continuous recalibration. The proposed “multi-hop” calibration concept represents the main contribution of this paper. The method relies on the proximity of devices which makes them “collocated” just enough that the machine learning algorithms could use one as a reference for the other for general calibration purposes, but would still have subtle differences because of the raw uncalibrated measurements of each device that are used as input to the trained machine learning algorithms. This method would enable a network of devices that could continuously calibrate each other while still having the ability to be sensitive to their local pollutant occurrences. The observed pollutants (NO_2_ and PM_10_) are measured with different sensor types (chemical and optical), and to the best of the authors knowledge, are not present in current hybrid network calibration transfer research. This also represents an important contribution of the paper. We show that the proposed “multi-hop” calibration concept is successful, thus, representing a very promising solution that could be used as the basis of a highly economic, in both installation and maintenance aspects, air quality monitoring platform that would be attractive to the governments of many cities.

The paper is organized as follows: In Section 2, the used measurement devices and initial calibration are presented. The hybrid sensor network concept is described, as well as the calibration transfer concept. In Section 3, the performance evaluation results are presented, including a discussion about the achieved results and paper contribution. Finally, Section 4 concludes the paper and provides future work directions.

## 2. Materials and Methods

### 2.1. Measurement Devices

Currently, there are many low-cost devices available on the market, with different performances, depending on which sensors (measurement range, accuracy, precision) and transmission technology (GPRS, 3G, LTE, NB-IoT, WiFi, LoRa, BLE) they use. Based on the manufacturer’s available data, these devices are, on average, 25 times cheaper than public monitoring reference stations equipped with a set of sensors that measure the same pollutants. In this study, 10 DunavNET ekoNET devices AQ10x (DunavNet, Novi Sad, Serbia) [25] for outdoor air quality monitoring were used for measurements and data collection in Novi Sad, Serbia. Each device contained the following sensors: Alphasense NO_2_-B43F gas sensor (Alphasense. New York, NY, USA) (measurement range: 0–20 ppm, unit μg/m^3^, accuracy ± 2% FS), Plantower PMS7003 optical counters PM_10_ (Plantower, Beijing, China) (measurement range: 0~1000 μg/m^3^, accuracy ± 2% FS), and Bosch BME 280 (Bosch, Gerlingen, Germany) air temperature (T) and relative humidity (RH) sensors. Measurements were collected with a 1 min time resolution and transferred via GPRS to a cloud database—Microsoft Azure (Microsoft, Redmond, WA, USA), where the measurements were processed and stored. Additionally, data were also collected from a single public air quality Automatic Monitoring Station run by the Serbian Environmental Protection Agency (SEPA) located in the city of Novi Sad, which was used as a reference monitoring station in this paper. Devices were deployed at a height of about 2 m.

### 2.2. Hybrid Sensor Network

**Figure 1:**
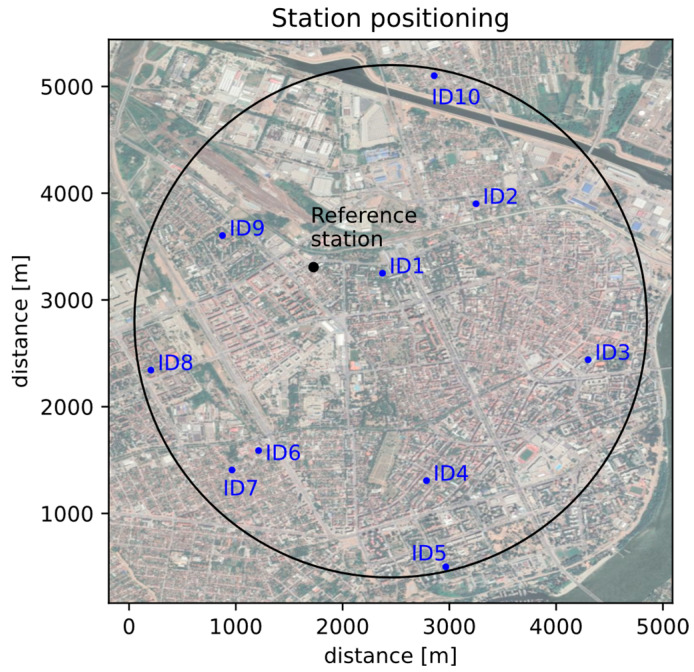
The spatial arrangement of measuring stations, Novi Sad, Serbia. ID1-ID10 represent the locations of the low cost devices used in the study.

Observed hybrid sensor network for air quality monitoring consisted of 10 AQ10x devices with the same configuration (NO_2_, PM_10_, T and RH sensors) and one SEPA monitoring reference station (Novi Sad, Rumenacka, urban traffic) equipped with the same set of sensors as the low-cost sensor network with AQ10x devices. In the reference station, PM_10_ was monitored by using Tapered Element Oscillating Microbalance (TEOM) technology. The network was installed in the city of Novi Sad, and Figure 1 shows the spatial arrangement of the installed devices with the low-cost sensors (marked ID1-ID10) and the reference monitoring station. The low-cost devices were installed and governed by PUC (Public Utility Company) Informatika, Novi Sad, Serbia. The devices were mounted in the courtyards of available public institutions, at least ten meters away from main roads (urban background). In the observed monitoring urban area, there are no specific sources of pollution, and the air quality is mostly affected by traffic and transport, dust, and domestic heating-related pollution.

The area covered by the hybrid sensor network was marked with a black circle with a half-diameter of 2.4 km. The public monitoring station was slightly dislocated in relation to the center of the imaginary circle, which did not significantly affect the analysis and general conclusions.

### 2.3. The Initial Calibration

As the first step in building a hybrid sensor network, initial calibration of all low-cost devices was conducted by using the data from a reference monitoring station. All devices were collocated for seven consecutive days in November. In this initial calibration phase, the most common method of linear regression calibration was used [26]. The calibration was performed for each individual low-cost sensor and for each pollutant separately, using the low-cost sensor measurements as inputs and the reference pollutant measurements as outputs. A simple algorithm was used in this step as the goal was to make the devices have a similar output value range so that recalibration could later be performed and would provide more accurate calibration models since a greater variety and quantity of data would be available. The coefficients obtained in each individual calibration were applied to the raw measurements of the corresponding low-cost device, and all further analysis was performed on these calibrated values. The commonly used and widely accepted metrics, RMSE (Root Mean Square Error) and Pearson correlation coefficient (r) [27] were later used to evaluate the recalibration performance.

**Figure 2:**
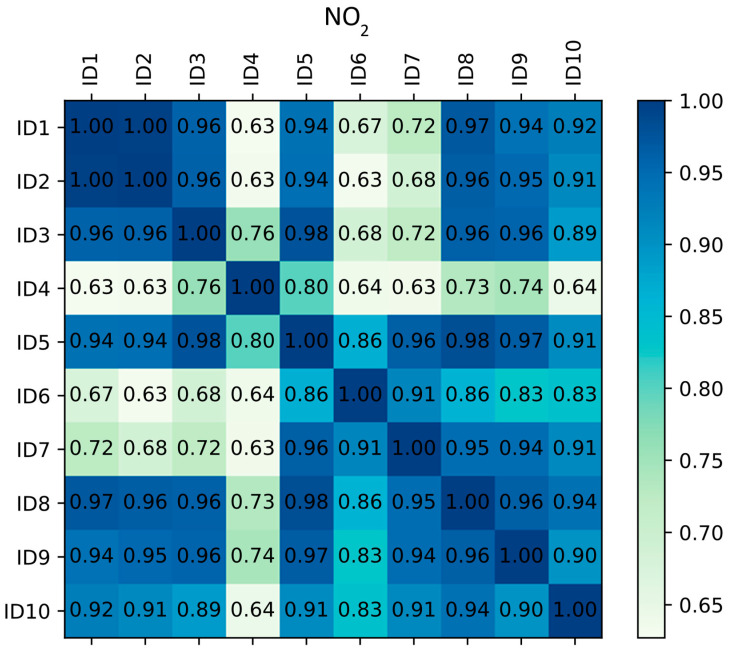
Correlation matrix of co-located low-cost devices for NO_2_.

**Figure 3:**
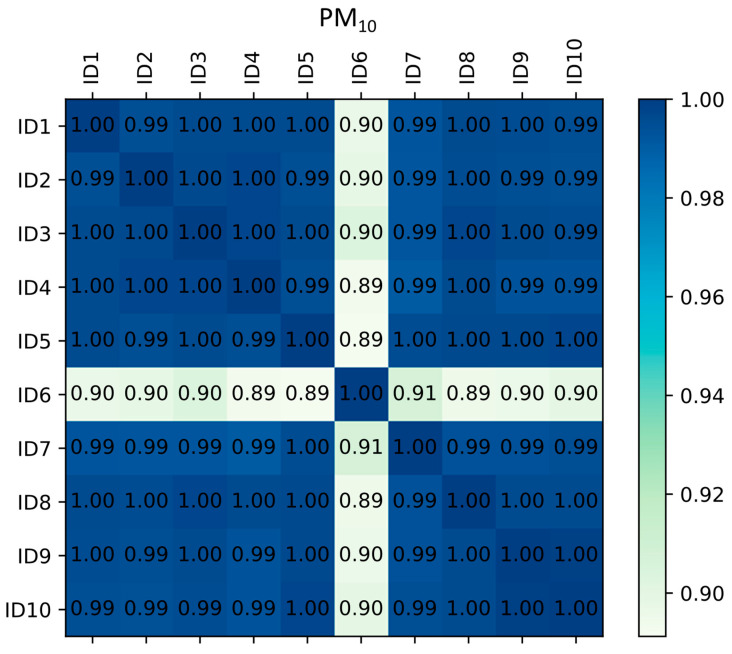
Correlation matrix of co-located low-cost devices for PM_10._

Since the measurement accuracy of every single low-cost sensor highly depends on the sensor’s chemical and physical characteristics, and having in mind that every sensor could have different measurements, it was of high interest to mutually compare the measurement correlation of observed devices. Although the measurement correction applied through the linear regression algorithm does correct the overall range of output values for the sensors, it does not influence the Pearson correlation coefficient. For this reason, the comparison was performed to see if the sensors followed similar trends. For data from the collocation period, correlation coefficients between measurements of different low-cost devices are shown in Figure 2 for NO_2_ and Figure 3 for PM_10_.

The agreement between the measuring devices for NO_2_ can be considered high, with the lowest correlation coefficient being 0.63. It is interesting to note, however, that the variation between measurement agreement does exist and that certain devices had a nearly perfect correlation (e.g., ID9 and ID10), while others do correlate, but to a lower degree. This indicates that to create a perfect correspondence between two low-cost sensors or a low-cost sensor and reference measurement for NO_2_, and non-linear correction would be necessary.

Unfortunately, after the initial sensor calibration, the NO_2_ sensor of the device ID8 malfunctioned, resulting in the lack of NO_2_ measurements from this device, which is why ID8 is omitted in the following section: *Results and Discussion of the Calibration Transfer Evaluation.*

A slightly different situation can be observed for PM_10_ measurements as compared to NO_2_. Although there were variations in the correlation, all stations except ID6 had a correlation coefficient of 0.99 or higher, which indicates a very high degree of conformity. Furthermore, the ID6 station, although it had the lowest correlation coefficients with other stations, still reached values of 0.89, which indicated a high degree of agreement. The agreement between relative humidity and temperature measurements of low-cost devices and the reference device was sufficiently high, which indicated good usability of the weather estimation capabilities of the low-cost devices without any need for corrections.

For the purposes of evaluating sensor degradation over time, we would suggest a short collocation period with a reference monitoring station on a yearly basis to ensure that the sensor sensitivity is sufficiently high.

### 2.4. The Concept of Calibration Propagation

After the initial collocation period, during which the initial calibration was performed using linear regression, the sensors were placed in real conditions, with a distance from the reference station ranging from 0.7 to 3 km of the reference station, with the spatial arrangement shown in Figure 1.

The concept of calibration propagation was based on placing low-cost stations in a “row”, where each station was calibrated based on the previous one, while the first low-cost station was chosen to be the closest to the reference one. In the context of a larger and more complex sensor network, the implementation would imply a matrix of the distance of each “low-cost” device to each, where the first would be determined as the closest to the reference station, and then every other low-cost device would be selected based on availability and distance from the available “reference” (i.e., calibrated station). In the order that was set for the validation of the solution, the ID of the station also indicated its order in the described list, and two “directions” of calibration were investigated. Direction 1 implied that station IDi was calibrated based on station IDi−1, for i greater than 1, and station ID1 was calibrated on the basis of reference. Direction 2 implied that ID10 was calibrated by the reference, while IDi was calibrated based on IDi+1 for i ranging from 9 to 1.

The calibration involved training a selected machine learning algorithm (ML) that solved the regression problem, i.e., mapping the input data of the ML algorithm (“low-cost” measurements and meteorological parameters–temperature and relative humidity) to the output data (measurements from the reference station). As part of the validation of the developed concept, the algorithm random forest (RF) was implemented and used for the stated calibration. Multiple ML algorithms could have been used at this step, but for the sake of the calibration propagation process, only RF was selected, based on the results of the authors’ previous work [24], and to make the analysis performed in this paper easier to interpret.

We will now give the explanation for the calibration propagation for direction 1, with direction 2 having the exact same principle, just the opposite order of calibration propagation.

**Figure 4:**
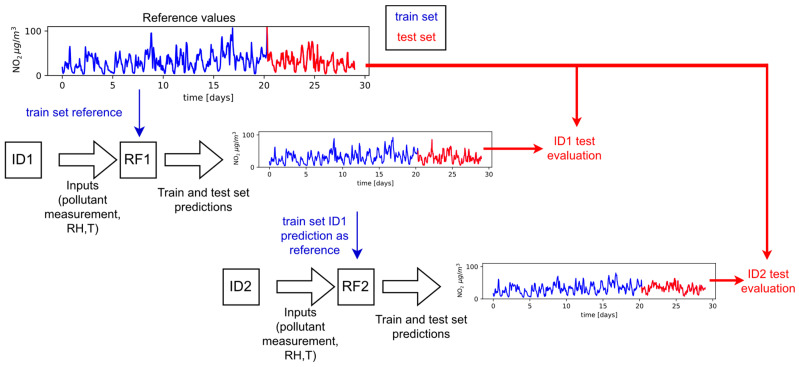
Illustration of the calibration propagation process and evaluation for devices ID1 and ID2.

Within the calibration propagation in direction 1, the algorithm RF1, which corresponds to station ID1, was first trained. This algorithm took low-cost measurements of the specific pollutant as input data, relative humidity and temperature, and it took the values of the observed pollutant measured from the reference measuring station as output data. Through the training process, the algorithm changed its parameters and “learned” to predict the output values based on the input values. In the next step of calibration propagation, the algorithm RF2 corresponding to station ID2 was trained, with the fact that the reference measurements were not taken from the reference measuring station but from station ID1 after calibration (output of algorithm RF1). This process continued until all low-cost stations were calibrated. The steps for calibration propagation performed for a single month for NO_2_, are illustrated for devices ID1 and ID2 in Figure 4.

As part of the performed algorithm validation, all “low-cost” stations were in the vicinity to the real reference station, so the results for each station in the evaluation period were obtained by comparison with the real reference station, but the idea was to show that, after calibration, low-cost stations can be used as a reference for other low-cost stations. The results obtained during the evaluation period included an increase in the r and a decrease in the RMSE parameter.

The concept of calibration propagation involved a chain transfer of calibration during the first three weeks of each month and an evaluation of the transferred calibration during the last week of the given month. Therefore, based on the data from the first 3 weeks during the observed month, a random forest algorithm was trained, which, based on the data from the first low-cost station (ID1), tried to predict the values measured at the reference measuring station. This procedure was performed separately for NO_2_ and PM_10_ measurements, but in both cases data on relative humidity and air temperature from low-cost devices were used. From this moment, for the further training of a separate algorithm for the second low-cost measuring station (ID2), the data from the measuring station ID1, corrected by the previously developed RF algorithm, were considered as reference values. In this way, using data from the first three weeks of the month, 19 RF algorithms were developed (one each for NO_2_ and PM_10_, for each low-cost station) where each station relied on corrected data from the previous one (in the order shown in Figure 1.), except for the first measuring station that relied directly on the reference one.

For the purposes of further analysis, the methodology was applied to data from two different months: February and May. These months were chosen as representatives of different seasons, and hourly measurements during these months were used to analyze the possibility of propagating calibration from device to device. For the analysis, only data points that contained valid measurement data from all sensors (low-cost pollutant, relative humidity, temperature and reference measurements) were included.

The processing of the data, as well as the ML algorithm implementation and data visualization, were completed in the Python programming language, using the sklearn and matplotlib libraries [28,29,30].

## 3. Results and Discussion of the Calibration Propagation Evaluation

Testing of the developed algorithms was performed on the data of the last week for evaluation that would be similar to a real application. The measurements from each low-cost measuring station, corrected by the appropriate RF algorithm, were compared with the measurements from the real reference station. The results obtained during the evaluation were presented through the increase of the r and a decrease in the RMSE parameter after calibration.

This process tests the effectiveness of chain calibration propagation from device to device, assuming that all stations are close enough to the reference for the purposes of this comparison. The ranges of measured variables for the reference station and low-cost stations (before calibration propagation) are given in Table 1.

### 3.1. February Results

**Figure 5:**
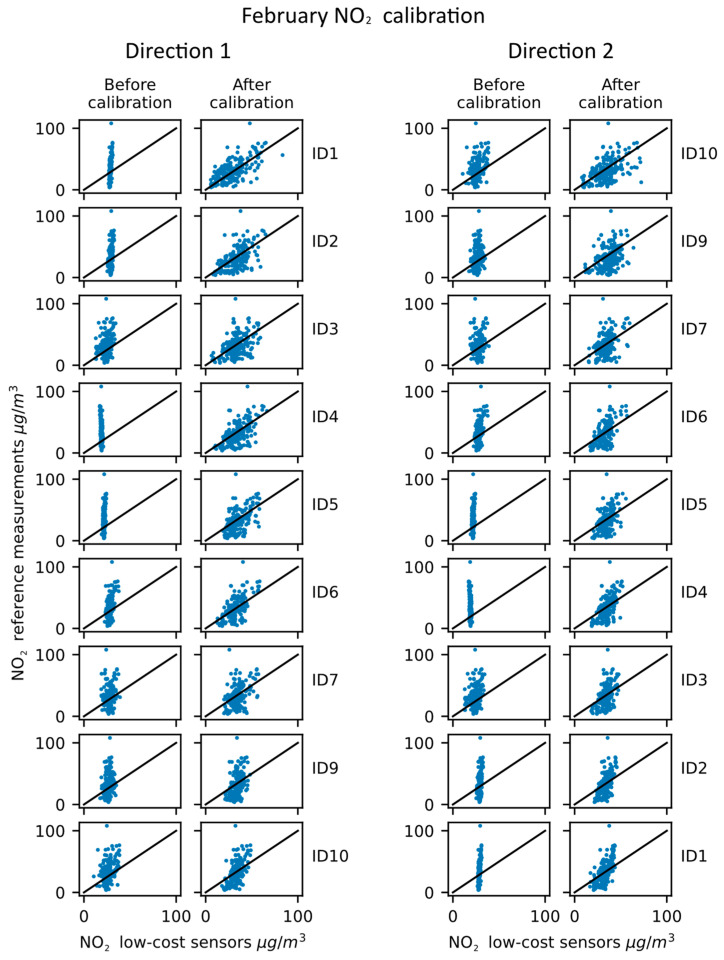
NO_2_ calibration propagation—February results.

The results of the calibration propagation evaluation performed in February are shown in Table 2 and in Figure 5.

The results shown in Table 2 indicate a clear improvement in the NO_2_ calibration propagation for both directions and for both the correlation coefficient and the RMSE. On the other hand, there is quite a prominent variability between the measurement improvements between the stations. The direction of calibration is also shown to influence the improvement of parameters, indicating that the order in which the calibration transfer is performed can indeed influence the results for individual stations, but the overall improvement was still present. The visual presentation of the calibrated data given in Figure 5 also illustrates the improvements quite well. Notably, the device ID1, ID2, ID4 and ID5 had quite a narrow output of values prior to the calibration, but is shown to have considerably improved for both directions of the calibration propagation.

**Figure 6:**
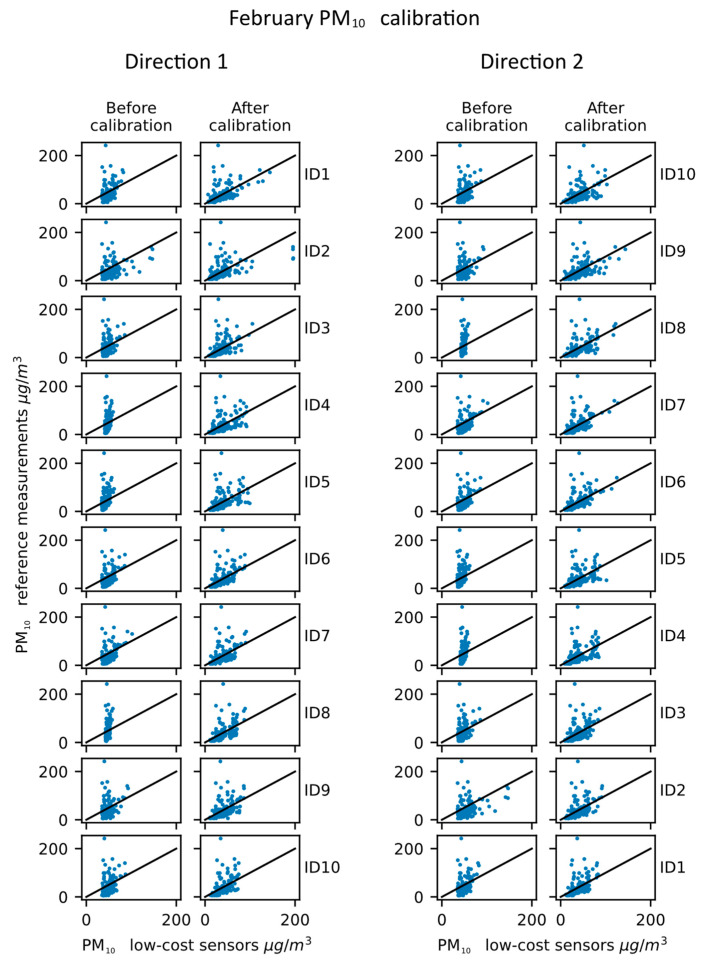
PM_10_ calibration propagation—February results.

The results obtained for the PM_10_ measurement calibration are shown in Table 3 and Figure 6.

The results obtained for the PM_10_ measurements in February draw similar conclusions as the ones for NO_2_. The improvements for each low-cost station are present, but they do vary from station to station. The correlation plots that are displayed in Figure 6. also show improvements, especially in the range of values that come as the output of the low-cost devices. The correlation between the calibrated sensor measurements and the reference ones can be observed to be higher for PM_10_ than for the NO_2_, which stands in line with the fact that sensors initially had a higher correlation between themselves for PM_10_ measurements than for the NO_2_ measurements.

### 3.2. May Results

**Figure 7:**
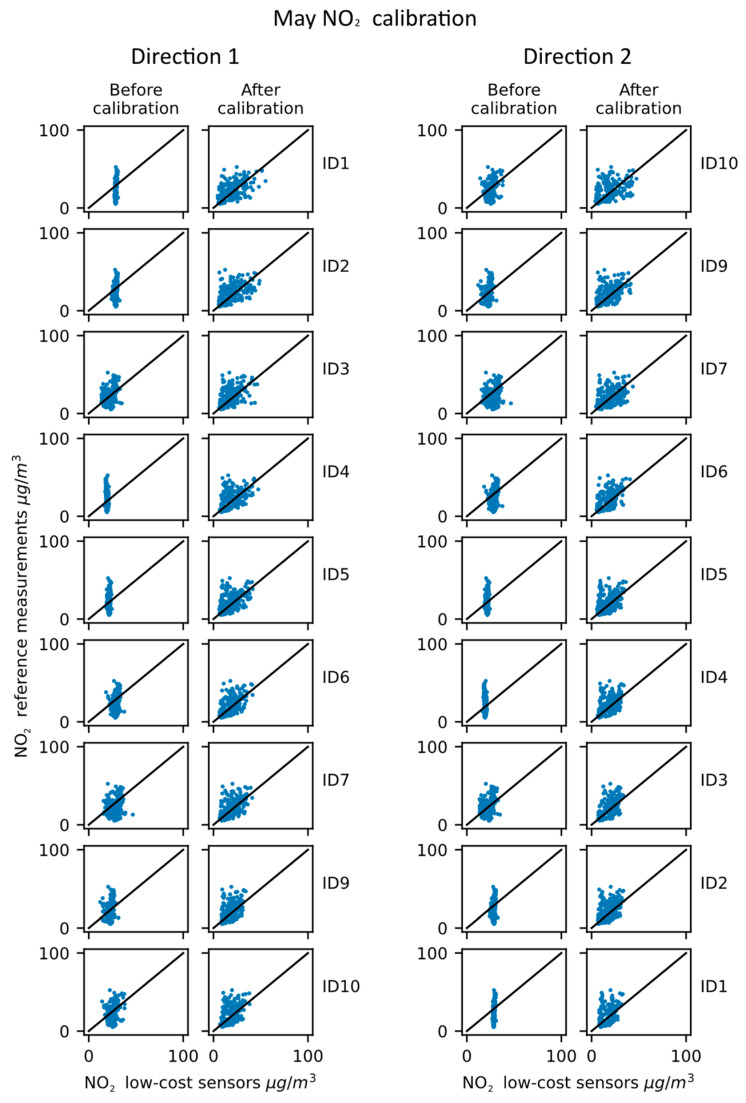
NO_2_ calibration propagation—May results.

The results obtained in the month of May, for NO_2_, are shown in Table 4 and in Figure 7.

The NO_2_ measurements in May show overall similar trends to the ones from February, with a couple of slight differences. The RMSE improvements are not always consistent, with the negative values indicating an increase in the parameter. The correlation of the sensors is, on the other hand, improved for all sensors except for sensor ID1 in direction 2. The output ranges of ID1, ID2, ID4 and ID5 are still quite narrow, and despite varying improvements in the metrics, it can be observed in the figure that their output range was widened. The lack of improvement of correlation of ID1, for direction 2, can also be observed in Figure 7, especially when comparing the results with the ones from direction 1.

**Figure 8:**
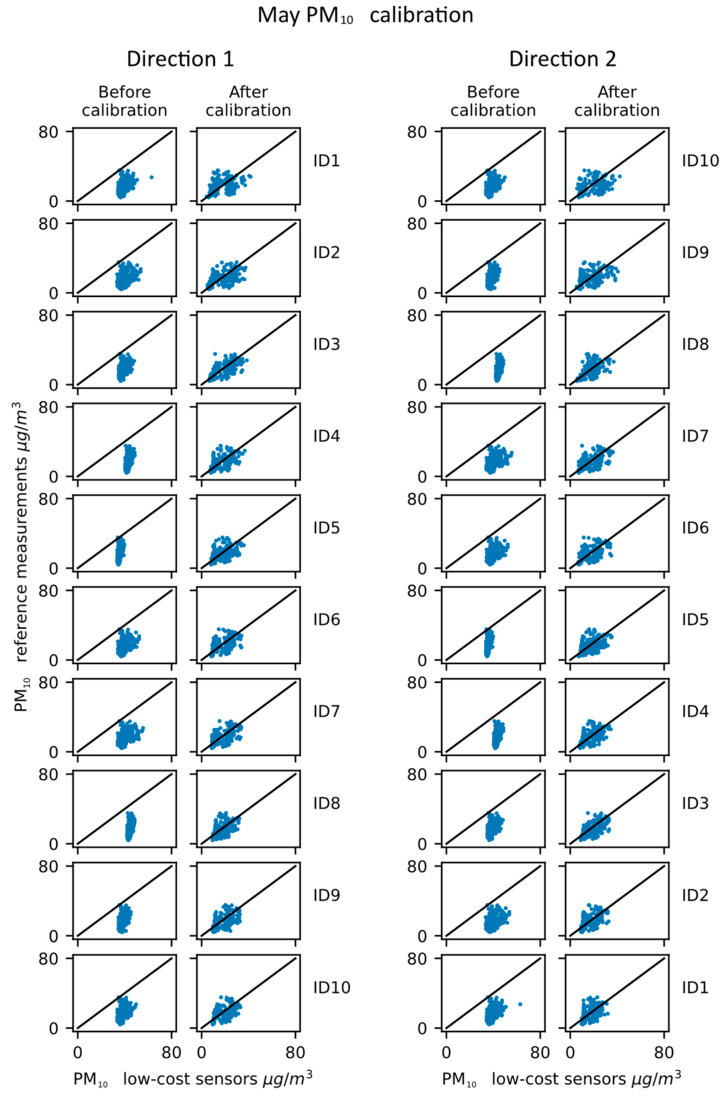
PM_10_ calibration propagation—May results.

Lastly, the results of the PM_10_ calibration propagation for May are given in Table 5 and Figure 8.

The results of the PM_10_ calibration in May show several differences in comparison to the February results. First, the correlation improvement is not present for all devices, with devices ID1, ID5 and ID8 having a decrease in the r value for both directions. The values of the PM_10_ are also much lower than they were in February, with the maximum values being around 80 $\text{µ}g/m^{3}$ in May, while the maximum values of PM_10_ in February were around 200 $\text{µ}g/m^{3}$. This is to be expected as PM_10_ emissions are higher in colder months due to the heating season. This could also be the reason for the differences in correlation performance. On the other hand, clear offsets for all sensors can be seen visually on the uncalibrated data in Figure 8., and the offsets are considerably improved after calibration, which is shown both in the RMSE parameter and on the visual presentation. The cause of this offset before the calibration propagation is probably the deterioration of sensors, as the PM_10_ sensors tend to have increased particle residue over time. The sensors measure the dispersion of laser light, which is caused by the particles, so the gathered particle residue could cause more laser dispersion and, therefore, an overestimation of values.

The presented data indicate several conclusions. First of all, there is a general improvement for the RMSE parameter for PM_10_ measurements, which indicates the successful operation of the developed algorithms and the successful chain transfer of the calibration. Additionally, it can be noted that in the month of February, there was a general noticeable improvement for both the r factor and the RMSE factor for all stations. For the month of May, improvement is mostly present, but not exactly for every metric and every device. An important conclusion is that although one of the devices does not benefit from calibration, it certainly does enable subsequent devices to be calibrated and thus does not break the chain.

The maximum improvement of the r factor, for a single low-cost device, for NO_2_ was 0.35 and for PM_10_ was 0.14. For the RMSE, the best results were a decrease of 6.82 $\text{µ}g/m^{3}$ for NO_2_ measurements and 20.56 $\text{µ}g/m^{3}$ for PM_10_ measurements. The summary of the obtained results in terms of the average values and standard deviations of r and RMSE for all low-cost devices can be seen in Table 6.

The results presented in Table 6 show that on average, the increase of r for NO_2_ was 34.2% and 58.6% for the two observed months, indicating a clear improvement, especially in May. The reduction of RMSE was not as prominent in terms of percentages as the r for NO_2_, but it is still present. For PM_10_, the improvements in terms of metric percentages are reversed. The r value has an improvement for both months, but it is smaller than NO_2_. The decrease of the average PM_10_ RMSE value is comparable to the NO_2_ decrease in February, but in May the decrease of the RMSE value was quite substantial (67.4%). Considering the deterioration that can occur in low-cost sensors, a benefit for both electrochemical (NO_2_) and optical (PM_10_) sensor can be obtained, as shown by the data displayed in Table 6.

The concept implemented in this paper relies on the fact that ML algorithms (in this case, RF) learn to predict data on the train set quite well, but if they are implemented with enough data and with the appropriate amount of complexity, they will not overfit. Overfitting implies that the algorithm adapts the parameters to the data on the training set too much, rendering it useless when obtaining predictions for data points out of the training set. If the algorithms are implemented properly, the concept proposed in this paper assumes that with a grid of sensors that is dense enough (approximately one device for every $2\;{\mathrm{km}}^{2}$ in this study), two adjacent sensors can be effectively collocated, and one can be used to calibrate another. By ever so slightly changing the reference values through the calibration propagation chain (from the initial reference to the RF predictions of the appropriate sensors), we believe that over time, the spatial differences between the sensors will be accounted for. The RF algorithms will learn to correct the measurements in terms of RH and temperature, but will take into account local changes that occur due to them being present in the RF training process of their predecessor. This offers a method for calibration that would not require any moving of the devices and is fundamentally a different concept from the ones present in the literature [17,21]. The drawback of the evaluation of the proposed method in this paper is that all low-cost stations were in the vicinity of a single reference station. The test which would test our hypothesis in a way that would be more similar to real-world applications is having at least two reference stations connected by a long chain of low-cost devices. However, the initial concept of propagating the calibration in a circle around the reference station has shown promising results (with two different directions considered) and could possibly be used in combination with other calibration transfer methods [17,21] to provide a more stable system.

## 4. Conclusions

The proposed solution introduces a new method for calibration propagation of low-cost sensors in a hybrid network consisting of a reference measuring station and a larger number of devices with low-cost sensors, which has not been sufficiently analyzed in the available literature, especially for the NO_2_ and PM_10_ measurements. After the application of the proposed calibration propagation method, there was an improvement in the RMSE parameter for the measurements, which indicates the successful operation of the developed algorithms and the successful chain transfer of the calibration. Another result of the applied method was an overall improvement of the r coefficient. An important conclusion is that although one of the devices did not benefit from calibration, it certainly successfully enabled subsequent devices to be calibrated and thus did not break the chain. The proposed method can be effectively used in a hybrid sensor network in general for sensor calibration and recalibration, and if one of the sensors begins to show signs of inaccuracy, recalibration can be performed by correlation with a reference station or cross-calibration compared to other recently recalibrated devices in that area. By applying this method, significant savings could be achieved during the sensor recalibration process since it is not necessary to physically move the devices to the measuring station or to a laboratory in order to perform the sensor recalibration. In addition, by enabling large-area coverage with low-cost sensors through the proposed calibration transfer method (propagating through a chain of low-cost devices), a lower number of expensive reference stations can be used, and a denser network consisting of only low-cost devices could be implemented with increased reliability.

The directions for future work would include observing other pollutants and analyzing larger network systems, including the analysis of the necessary sensor grid density for various pollution monitoring applications. Developing and testing the pollution map creation potential would also be of interest, as well as implementing calibration algorithms that do not rely on single point measurements, but rather on sequences of measurements, focusing particularly on convolutional neural networks.

## Figures and Tables

**Table 1:** Measurement ranges in $µg/m^{3}$ (average ± standard deviation) for NO_2_ and PM_10_.

Device ID	February		May	
	NO_2_	PM_10_	NO_2_	PM_10_
Ref.	31.5 ± 18.1	37.7 ± 31.0	21.3 ± 12.5	21.6 ± 11.3
ID1	28.7 ± 1.0	49.4 ± 12.4	28.5 ± 0.8	40.7 ± 4.8
ID2	29.3 ± 1.5	55.5 ± 19.2	28.1 ± 2.0	41.6 ± 5.8
ID3	23.9 ± 4.4	47.1 ± 11.2	22.5 ± 4.6	39.5 ± 3.4
ID4	18.9 ± 0.7	47.3 ± 4.8	19.6 ± 0.8	43.4 ± 2.1
ID5	21.8 ± 0.8	42.8 ± 6.8	21.2 ± 1.2	35.8 ± 1.7
ID6	28.1 ± 2.7	49.8 ± 12.5	28.1 ± 3.2	41.2 ± 5.1
ID7	27.5 ± 3.3	52.2 ± 15.7	27.1 ± 4.8	42.1 ± 5.8
ID8	/	47.7 ± 4.3	/	44.7 ± 1.9
ID9	26.2 ± 3.1	46.9 ± 11.1	22 ± 3.3	39.3 ± 3.6
ID10	26.0 ± 4.5	49.9 ± 13.4	24.5 ± 4.8	39.0 ± 3.7

**Table 2:** Results of the NO_2_ calibration propagation in February, improvements in comparison to the parameters before calibration.

Device ID	r Increase		RMSE $\left[µg/m^{3}\;\right]$ Decrease	
	Direction 1	Direction 2	Direction 1	Direction 2
ID1	0.01	0.01	3.27	2.54
ID2	0.15	0.20	1.90	1.84
ID3	0.12	0.21	1.96	2.77
ID4	0.18	0.17	6.82	6.58
ID5	0.11	0.06	4.16	3.69
ID6	0.06	0.03	2.47	1.95
ID7	0.24	0.22	1.34	1.14
ID9	0.17	0.16	1.22	0.45
ID10	0.17	0.13	1.97	0.96

**Table 3:** Results of the PM_10_ calibration propagation in February, improvements in comparison to the parameters before calibration.

Device ID	r Increase		RMSE $\left[µg/m^{3}\right]$ Decrease	
	Direction 1	Direction 2	Direction 1	Direction 2
ID1	0.06	0.06	3.33	3.76
ID2	0.07	0.10	3.02	6.52
ID3	0.08	0.09	3.05	3.27
ID4	0.04	0.05	3.91	4.09
ID5	0.05	0.04	2.11	1.72
ID6	0.08	0.11	4.19	4.67
ID7	0.06	0.09	4.10	4.71
ID8	0.05	0.06	5.27	5.05
ID9	0.09	0.14	3.05	3.25
ID10	0.11	0.14	4.20	4.74

**Table 4:** Results of the NO_2_ calibration propagation in May, improvements in comparison to the parameters before calibration.

Device ID	r Increase		RMSE $\left[µg/m^{3}\right]$ Decrease	
	Direction 1	Direction 2	Direction 1	Direction 2
ID1	0.10	−0.01	1.56	1.09
ID2	0.35	0.23	1.61	1.34
ID3	0.28	0.32	0.52	1.21
ID4	0.27	0.20	1.06	0.45
ID5	0.23	0.16	−0.28	−0.81
ID6	0.33	0.28	2.60	1.93
ID7	0.32	0.26	2.35	1.45
ID9	0.24	0.27	0.25	−0.2
ID10	0.17	0.13	0.72	−1.02

**Table 5:** Results of the PM_10_ calibration propagation in May, improvements in comparison to the parameters before calibration.

Device ID	r Increase		RMSE $\left[µg/m^{3}\right]$ Decrease	
	Direction 1	Direction 2	Direction 1	Direction 2
ID1	−0.08	−0.08	14.33	15.76
ID2	0.03	0.03	15.36	16.76
ID3	0.11	0.13	14.90	15.45
ID4	0.00	0.02	18.73	19.25
ID5	−0.03	−0.05	11.47	11.64
ID6	0.10	0.10	16.22	16.16
ID7	0.12	0.10	17.19	16.90
ID8	−0.09	−0.07	20.56	20.25
ID9	0.04	−0.02	14.59	13.39
ID10	0.13	0.01	14.96	12.74

**Table 6:** The summary of the obtained results for all low-cost devices before and after calibration propagation.

Evaluation Month and Pollutant	r before Calibration (Mean ± std)	r after Calibration (Mean ± std)	% of r Increase	RMSE $[µg/m^{3}]\;$ before Calibration (Mean ± std)	RMSE$[µg/m^{3}]\;$ after Calibration (Mean ± std)	% of RMSE Decrease
NO_2_ Feb	0.38 ± 0.14	0.51 ± 0.09	34.2	17.64 ± 1.51	15.03 ± 0.90	14.8
PM_10_ Feb	0.43 ± 0.03	0.50 ± 0.03	16.3	31.39 ± 0.96	27.49 ± 0.94	12.4
NO_2_ May	0.29 ± 0.08	0.46 ± 0.05	58.6	11.43 ± 0.77	10.55 ± 0.58	7.7
PM_10_ May	0.38 ± 0.03	0.41 ± 0.06	7.9	23.50 ± 2.25	7.67 ± 0.62	67.4

## Data Availability

Restrictions apply to the availability of these data. Data was obtained from PUC Informatika and are available from the authors with the permission of PUC Informatika.

## References

[1] The United Nations Human Settlements Programme (UN-Habitat), World Cities Report 2022. https://unhabitat.org/sites/default/files/2022/06/wcr_2022.pdf.

[2] Directive 2008/50/EC of the European Parliament and of the Council of 21 May 2008 on Ambient Air Quality and Cleaner Air for Europe OJ L 152, 11.6.2008. https://eur-lex.europa.eu/legal-content/en/ALL/?uri=CELEX%3A32008L0050.

[3] Department of Ecology, State of Washington Air Monitoring Site Selection and Installation Procedure. https://apps.ecology.wa.gov/publications/documents/1602021.pdf.

[4] Greater London Authority, Guide for Monitoring Air Quality in London. https://www.london.gov.uk/sites/default/files/air_quality_monitoring_guidance_january_2018.pdf.

[5] Johnston S.J., Basford P.J., Bulot F.M.J., Apetroaie-Cristea M., Easton N.H.C., Davenport C., Foster G.L., Loxham M., Morris A.K.R., Cox S.J. (2019). City Scale Particulate Matter Monitoring Using LoRaWAN Based Air Quality IoT Devices. Sensors.

[6] Schneider P., Castell N., Vogt M., Dauge F.R., Lahoz W.A., Bartonova A. (2017). Mapping Urban Air Quality in near Real-Time Using Observations from Low-Cost Sensors and Model Information. Environ. Int..

[7] Popoola O.A.M., Carruthers D., Lad C., Bright V.B., Mead M.I., Stettler M.E.J., Saffell J.R., Jones R.L. (2018). Use of Networks of Low Cost Air Quality Sensors to Quantify Air Quality in Urban Settings. Atmos. Environ..

[8] Zaidan M.A., Xie Y., Motlagh N.H., Wang B., Nie W., Nurmi P., Tarkoma S., Petäjä T., Ding A., Kulmala M. (2022). Dense Air Quality Sensor Networks: Validation, Analysis, and Benefits. IEEE Sens. J..

[9] Chen F.-L., Liu K.-H. (2020). Method for Rapid Deployment of Low-Cost Sensors for a Nationwide Project in the Internet of Things Era: Air Quality Monitoring in Taiwan. Int. J. Distrib. Sens. Networks.

[10] Penza M., Suriano D., Pfister V., Prato M., Cassano G. Urban Air Quality Monitoring with Networked Low-Cost Sensor-Systems. Proceedings of the Eurosensors.

[11] Maag B., Zhou Z., Saukh O., Thiele L. (2017). SCAN: Multi-Hop Calibration for Mobile Sensor Arrays. Proc. ACM Interact. Mob. Wearable Ubiquitous Technol..

[12] Saukh O., Hasenfratz D., Thiele L. (2015). Reducing Multi-Hop Calibration Errors in Large-Scale Mobile Sensor Networks. Proceedings of the the 14th International Conference on Information Processing in Sensor Networks.

[13] Motlagh N.H., Petäjä T., Kulmala M., Trachoma S., Lagerspetz E., Nurmi P., Li X., Varjonen S., Mineraud J., Siekkinen M. (2020). Toward Massive Scale Air Quality Monitoring. IEEE Commun. Mag..

[14] Lin Y., Dong W., Chen Y. (2018). Calibrating Low-Cost Sensors by a Two-Phase Learning Approach for Urban Air Quality Measurement. Proc. ACM Interact. Mob. Wearable Ubiquitous Technol..

[15] Dong W., Guan G., Chen Y., Guo K., Gao Y. Mosaic: Towards City Scale Sensing with Mobile Sensor Networks. Proceedings of the 2015 IEEE 21st International Conference on Parallel and Distributed Systems (ICPADS).

[16] Xiang Y., Piedrahita R., Dick R.P., Hannigan M., Lv Q., Shang L. (2013). A Hybrid Sensor System for Indoor Air Quality Monitoring. Proceedings of the Proceedings-IEEE International Conference on Distributed Computing in Sensor Systems, DCoSS 2013.

[17] Cheng Y., He X., Zhou Z., Thiele L. (2019). ICT: In-Field Calibration Transfer for Air Quality Sensor Deployments. Proc. ACM Interact. Mob. Wearable Ubiquitous Technol..

[18] Yan K., Zhang D. (2016). Calibration Transfer and Drift Compensation of E-Noses via Coupled Task Learning. Sens. Actuators B Chem..

[19] Zhang L., Tian F., Kadri C., Xiao B., Li H., Pan L., Zhou H. (2011). On-Line Sensor Calibration Transfer among Electronic Nose Instruments for Monitoring Volatile Organic Chemicals in Indoor Air Quality. Sensors Actuators B Chem..

[20] Fonollosa J., Fernández L., Gutiérrez-Gálvez A., Huerta R., Marco S. (2016). Calibration Transfer and Drift Counteraction in Chemical Sensor Arrays Using Direct Standardization. Sens. Actuators B Chem..

[21] deSouza P., Kahn R., Stockman T., Obermann W., Crawford B., Wang A., Crooks J., Li J., Kinney P. (2022). Calibrating Networks of Low-Cost Air Quality Sensors. Atmos. Meas. Tech..

[22] Barkjohn K.K., Gantt B., Clements A.L. (2021). Development and Application of a United States Wide Correction for PM(2.5) Data Collected with the PurpleAir Sensor. Atmos. Meas. Tech..

[23] Drajic D.D., Gligoric N.R. (2020). Reliable Low-Cost Air Quality Monitoring Using Off-The-Shelf Sensors and Statistical Calibration. Elektron. Ir Elektrotech..

[24] Vajs I., Drajic D., Gligoric N., Radovanovic I., Popovic I. (2021). Developing Relative Humidity and Temperature Corrections for Low-Cost Sensors Using Machine Learning. Sensors.

[25] Air Monitoring–EkoNET. https://ekonet.solutions/air-monitoring/.

[26] Engelhardt M., Bain L. (2000). Introduction to Probability and Mathematical Statistics.

[27] Teh H.Y., Kempa-Liehr A.W., Wang K.I.-K. (2020). Sensor Data Quality: A Systematic Review. J. Big Data.

[28] Van Rossum G., Drake F.L. (2009). Python 3 Reference Manual.

[29] Pedregosa F., Varoquaux G., Gramfort A., Michel V., Thirion B., Grisel O., Blondel M., Prettenhofer P., Weiss R., Dubourg V. (2011). Scikit-Learn: Machine Learning in {P}ython. J. Mach. Learn. Res..

[30] Hunter J.D. (2007). Matplotlib: A 2D Graphics Environment. Comput. Sci. Eng..

